# Modern Approaches to Rectal Cancer: Integrating Endoscopic, Surgical, and Oncological Care

**DOI:** 10.3390/cancers17172820

**Published:** 2025-08-28

**Authors:** Jiří Kotek, Jiří Cyrany, Miroslav Sirový, Pavel Novotný, Jiří Páral

**Affiliations:** 1 Department of Surgery, University Hospital Hradec Králové, 50005 Hradec Králové, Czech Republic; miroslav.sirovy@fnhk.cz (M.S.); pavel.novotny1@fnhk.cz (P.N.); jiri.paral@fnhk.cz (J.P.); 2 Department of Surgery and Gastroenterology, Faculty of Medicine in Hradec Králové, Charles University, 11636 Prague, Czech Republic; jiri.cyrany@fnhk.cz; 3 2nd Department of Internal Medicine—Gastroenterology, University Hospital Hradec Králové, 50005 Hradec Králové, Czech Republic; 4 Department of Field Surgery, Military Faculty of Medicine, University of Defence, 66210 Brno, Czech Republic

**Keywords:** rectal cancer, neoadjuvant therapy, endoscopic treatment, total mesorectal excision, minimally invasive surgery, pelvic exenteration, organ preservation, transanal surgery

## Abstract

Rectal cancer remains a significant clinical challenge due to its complex anatomy and the critical need to balance oncological radicality with functional preservation. Multimodal treatment strategies, including neoadjuvant therapy, advanced endoscopic techniques, and precise surgical approaches, have evolved to optimize patient outcomes. Neoadjuvant chemoradiotherapy improves resectability and local control in locally advanced tumors, while endoscopic treatment offers organ-preserving options for carefully selected early-stage cancers. Surgical resection, primarily through total mesorectal excision (TME), remains the cornerstone of curative therapy, with minimally invasive and transanal approaches enhancing precision and recovery. In advanced and recurrent cases, extended procedures such as pelvic exenteration provide potential for cure despite substantial morbidity. This review summarizes current evidence on the indications, techniques, and outcomes of neoadjuvant, endoscopic, and surgical treatments for rectal cancer, emphasizing individualized treatment planning to achieve optimal oncological and functional results.

## Simple Summary

Rectal cancer is a complex disease that requires a multidisciplinary and individualized treatment approach. Advances in endoscopic, surgical, and oncologic techniques have enabled more effective and less invasive strategies, particularly for early-stage tumors. Organ-preserving approaches are increasingly used in selected patients to avoid unnecessary radical surgery and preserve function. For locally advanced disease, neoadjuvant therapy plays a key role; however, recent studies suggest that some patients—especially those with certain T3 tumors—may be overtreated. This narrative review outlines current treatment options, including endoscopic resection, total mesorectal excision, and non-operative strategies, and emphasizes the importance of balancing oncological safety with quality of life. By synthetizing recent evidence, this review aims to support clinical decision-making and encourage a more personalized and judicious use of both surgical and non-surgical therapies in rectal cancer management.

## 1. Introduction

Colorectal cancer (CRC) represents one of the major oncological burdens of modern times. According to the most recent estimates from IARC GLOBOCAN (2022) (https://gco.iarc.fr/today/en/fact-sheets-cancers, accessed on 28 August 2025)., approximately 1.926 million new CRC cases and 904,000 related deaths were reported worldwide, making CRC the third most commonly diagnosed malignancy and the second leading cause of cancer-related mortality globally. Rectal cancer accounts for approximately one-third of all CRC cases. A significant demographic disparity is evident—regions with a very high Human Development Index (HDI), such as Europe, Australia, and North America, report the highest incidence and mortality rates. In contrast, sub-Saharan Africa and South Asia have substantially lower rates [1,2].

Over recent decades, a fundamental shift in the age profile of CRC has emerged, with a marked rise in incidence among adults younger than 50 years, referred to as early-onset colorectal cancer (EOCRC). An analysis of Global Burden of Disease (GBD) data from 1990 to 2021 confirms that the number of newly diagnosed EOCRC cases has nearly doubled globally. This increase is not confined to Western countries; it is observed worldwide, including in Eastern Europe, Asia, South America, and the Caribbean. The highest incidence of EOCRC is reported in high-HDI countries—Australia, the USA, South Korea, and New Zealand. By 2030, it is estimated that individuals under 50 may account for up to 23% of all rectal cancer cases [3,4]. Several professional oncology societies have already responded to this trend by lowering the recommended age for screening colonoscopy from 50 to 45 years [5].

The therapeutic approach to rectal cancer is inherently multimodal and necessitates comprehensive, multidisciplinary care. An integrated team comprising gastroenterologists, surgeons, oncologists, pathologists, and other specialists such as radiologists, anesthesiologists, and psychologists is essential. Treatment in certified comprehensive cancer centers, where interdisciplinary collaboration is standard, has been shown to significantly improve oncological outcomes and reduce recurrence rates.

The diagnostic workup for rectal cancer includes digital rectal examination, colonoscopy with histological verification, biomarker testing (CEA, CA 19-9), staging using thoracoabdominal CT and pelvic MRI, often supplemented by rectal endoscopic ultrasound. Genetic marker analysis in tissue samples—KRAS, NRAS, BRAF mutations; DPYD status; and dMMR/MSI status—is crucial for personalizing targeted therapies, including immunotherapy for MSI-high tumors.

Liquid biopsy has recently emerged as a promising, minimally invasive method for detecting and monitoring colorectal cancer. By analyzing circulating biomarkers such as ctDNA, CTCs, and exosomal RNAs, it offers potential applications in early diagnosis, assessment of minimal residual disease, real-time monitoring of treatment response, and early identification of recurrence. Although still under clinical validation, liquid biopsy may complement conventional diagnostic tools and help guide more personalized therapeutic strategies in both localized and metastatic disease [6].

Disease staging according to the UICC/TNM system guides the structure of therapeutic strategies [7]. Prognosis is strongly influenced by the timing of diagnosis. When treatment begins in early stages (T1–2 N0), the 5-year survival rate exceeds 90% [8,9], whereas cases with nodal involvement drop to approximately 40% [8,10], and those with distant metastases to 10–30% [11]. Therefore, optimizing screening, diagnosis, and multimodal treatment is essential to maximize patient benefit. In locally advanced cases, neoadjuvant chemoradiotherapy is followed by surgery, restaging, and possibly adjuvant therapy. The presence of distant metastases necessitates systemic chemotherapy, targeted therapies, and immunotherapy tailored to the tumor’s molecular profile.

## 2. Multidisciplinary Team

Designing an optimal treatment plan for patients with rectal cancer is a complex task that must align with the most recent guidelines of professional societies while also considering the patient’s overall health status and personal preferences. A multidisciplinary tumor board should always include an oncologist, surgeon, gastroenterologist, radiologist, and pathologist, with additional specialists such as an anesthesiologist, internist, or psychologist involved as needed. This team is responsible for carefully balancing risks and benefits to establish the most appropriate therapeutic strategy for each individual patient. Based on our institutional experience, all cases of rectal cancer that cannot be safely managed endoscopically, or in which endoscopic treatment has failed to achieve radicality, should be presented at the tumor board. Multidisciplinary tumor boards have become an important asset for the management and treatment of patients with rectal cancer.

## 3. Oncological Treatment

### 3.1. Neoadjuvant Therapy

Neoadjuvant therapy represents an integral part of the multimodal treatment of patients with locally advanced rectal cancer, particularly in cases of clinical stage cT3–4 or N+, or tumors with high risk of recurrence (e.g., extramural vascular invasion [EMVI], positive mesorectal circumferential resection margin [mrCRM], low-lying tumors, or lateral lymph nodes) [12,13].

In recent years, total neoadjuvant therapy (TNT) has gained widespread adoption with the goal of improving oncological outcomes, particularly systemic disease control and the rate of pathological complete response (pCR). TNT involves the sequential administration of radiotherapy and full-dose systemic chemotherapy prior to surgery. This approach is currently preferred in most high-volume oncology centers, as demonstrated by key trials such as RAPIDO, PRODIGE-23, OPRA, and STELLAR [13,14,15,16].

### 3.2. Radiotherapy

Radiotherapy plays a crucial role in the neoadjuvant treatment of locally advanced rectal cancer (LARC), particularly in patients with cT3–4, cN+, positive mrCRM, or EMVI. Standard protocols include long-course radiotherapy and short-course radiotherapy.

Long-course radiotherapy (LCRT) consists of administering 45–50.4 Gy in fractions of 1.8–2 Gy over 5–5.5 weeks, concurrently with fluoropyrimidines (continuous infusion of 5-FU or oral capecitabine). This combination acts as a radiosensitizer and is especially indicated for low-lying tumors where downstaging and sphincter preservation are critical.

The potential benefit of adding oxaliplatin has been discussed in the literature. The CAO/ARO/AIO-04 trial demonstrated a modest increase in pCR at the cost of increased toxicity, particularly hematologic and gastrointestinal [17].

Short-course radiotherapy (SCRT) involves administering 25 Gy in five fractions over one week without concurrent chemotherapy. Traditionally, surgery followed within 7–10 days, but recent TNT trials (e.g., RAPIDO, STELLAR) implemented delayed surgery with consolidation chemotherapy, thereby effectively incorporating SCRT into the TNT strategy [14,16,18].

In the RAPIDO trial, short-course radiotherapy followed by preoperative CAPOX or FOLFOX was associated with a higher pathological complete response rate of 27.7% compared to 13.8% with conventional chemoradiotherapy, as well as a reduction in the incidence of distant metastases [14]. SCRT may therefore be indicated in patients requiring a rapid therapeutic response or in those whose general condition does not allow them to tolerate long-course chemoradiotherapy. Compared to LCRT, SCRT offers a shorter overall treatment duration while providing adequate systemic coverage. A potential drawback, however, is the lower rate of local downstaging.

### 3.3. Chemotherapy

Chemotherapy may be delivered either as part of classic concurrent chemoradiotherapy (CRT) or as sequential systemic treatment within the TNT framework.

Classic CRT consists of the concurrent administration of a fluoropyrimidine (capecitabine or 5-FU) during LCRT. The addition of oxaliplatin has been evaluated in several studies (e.g., CAO/ARO/AIO-04), resulting in modest improvement in pCR but with increased toxicity [17]. Surgery typically follows 6–8 weeks after CRT. Adjuvant chemotherapy is selectively indicated based on histopathological findings.

Total neoadjuvant therapy (TNT) is based on the use of radiotherapy (SCRT or LCRT) combined with systemic chemotherapy (e.g., FOLFOX, CAPOX, or in some cases, FOLFIRINOX). Chemotherapy may be delivered prior to radiotherapy (induction) or after (consolidation). The RAPIDO and PRODIGE-23 trials demonstrated that TNT improves pCR rates (up to 28% vs. 14% with CRT), reduces the occurrence of distant metastases, and improves disease-free survival (DFS) [13,14]. A major advantage of TNT is the possibility of including patients with clinical complete response (cCR) in organ-preserving “watch-and-wait” strategies [19]. Table 1 summarizes the TNT and classic CRT differences.

Current guidelines from leading societies (ESMO, ASTRO, NCCN, ASCO) prefer TNT in cases of locally advanced rectal cancer (cT3–4, cN+, or high-risk features) [20,21,22,23]. TNT includes either SCRT or LCRT followed by systemic chemotherapy (e.g., FOLFOX, CAPOX) before surgery. Results from RAPIDO, PRODIGE-23, and STELLAR demonstrated statistically significant improvements in DFS and reductions in distant metastases, see Table 2 [13,14,16].

ASTRO emphasizes that TNT is the standard for cT3–4 and N+ disease, with the choice between SCRT with consolidation or LCRT with induction or consolidation chemotherapy [20]. The OPRA trial also supports the “watch-and-wait” approach in patients with cCR, showing that rectal continuity was preserved in up to 54% of conservatively managed patients [15].

The PROSPECT study showed noninferiority of neoadjuvant FOLFOX with selective CRT versus CRT alone. The study evaluated preoperative oncological treatment in patients with locally advanced rectal cancer (T2N1, T3N0, T3N1). Results of this trial observed were comparable overall survival and local recurrence rate. These results seemed to aim to reduce the radiotherapy preoperatively and postpone it to adjuvant treatment plan [24]. Despite the results, there was study evaluating pathological outcomes in real-world data supporting the previous finding in similar overall survival. Patients undergoing preoperative FOLFOX who omitted CRT had lower chance for R0 resection and pCR compared to patients with CRT alone [25].

### 3.4. Adjuvant Therapy

Adjuvant chemotherapy is indicated after total mesorectal excision (TME) in patients who did not receive TNT or had suboptimal resection (R1 or Dworak ≤ 2). The greatest benefit is seen in patients with higher pathological stages (ypT3/4, ypN+), particularly in the prevention of systemic recurrences.

Recommended regimens include oxaliplatin-based combination therapy (mFOLFOX6 or CAPOX). Three-month treatment duration is appropriate for patients with lower-risk disease (T1–3, N1), while six months is recommended for high-risk patients (T4/N2 or other adverse factors). The IDEA trial supported the non-inferiority of 3-month CAPOX in lower-risk patients [26]. An alternative regimen for patients who are intolerant to oxaliplatin—due to factors such as pre-existing peripheral neuropathy, advanced age, or significant comorbidities—is the de Gramont regimen. This protocol consists of leucovorin infusion followed by a continuous two-day infusion of 5-FU. The treatment is administered over 12 cycles, each repeated every 14 days, resulting in a total treatment duration of six months [13,27].

Adjuvant chemotherapy should ideally be initiated as early as possible postoperatively. The optimal timing is within 6–8 weeks. The GRECCAR-6 trial confirmed that extending the interval to 7–11 weeks does not worsen 5-year oncological outcomes [28]. However, retrospective analyses suggest that delays beyond 12 weeks are associated with reduced overall survival [29]. When planning treatment, the outcome of prior TNT, postoperative complications, and the patient’s overall condition must be considered.

### 3.5. Immunotherapy

Immunotherapy represents a novel and highly specific treatment option for rectal cancer patients, particularly in those with tumors characterized by high microsatellite instability (MSI-high) or deficient mismatch repair (dMMR). These tumors have a high mutational burden and neoantigen load, increasing immunogenicity and enabling effective use of immune checkpoint inhibitors, particularly anti-PD-1 antibodies (e.g., pembrolizumab, nivolumab) [30,31].

Clinical studies have demonstrated the remarkable efficacy of immunotherapy in metastatic MSI-high/dMMR colorectal cancers, with some patients achieving durable remission and improved survival [32,33]. Current research is exploring the use of immunotherapy in the neoadjuvant setting for locally advanced rectal tumors, with early data suggesting the potential for complete pathological responses and reduced need for radical surgery [34]. Implementation of immunotherapy into clinical practice requires precise histologic and molecular determination of MSI/MMR status.

### 3.6. Watch and Wait Strategy

In recent years, the “watch-and-wait” (W&W) approach has emerged as an alternative for patients with locally advanced rectal cancer who achieve a clinical complete response (cCR) following neoadjuvant chemoradiotherapy or total neoadjuvant therapy. This strategy aims to preserve the rectum and avoid morbidity from radical surgery, particularly permanent stoma formation and impaired functional outcomes. Evidence from the International Watch & Wait Database (IWWD), encompassing over 1000 patients, reports a 2-year cumulative local regrowth incidence of 25.2%, with salvage surgery being feasible in the majority of cases, and 5-year overall survival around 85% and disease-specific survival 94% [35]. A recent network meta-analysis demonstrated no significant differences in 5-year overall survival between W&W and radical surgery, supporting the safety of W&W in selected patients [36]. Another meta-analysis found comparable long-term oncological outcomes—including distant metastasis, disease-free survival, and overall survival—between W&W and radical surgery, although W&W showed a higher local recurrence rate but significantly lower permanent stoma rates [37]. Despite these encouraging results, the lack of standardized criteria for cCR assessment and follow-up protocols remains a significant limitation. Future research should focus on refining imaging tools and biomarkers to identify ideal candidates for W&W, and on establishing validated surveillance regimens to ensure safe organ preservation.

At our institution, follow-up of patients with cCR after TNT is performed as follows: pelvic MRI and sigmoidoscopy every 3–4 months during the first two years, then every 4–6 months until year five. Chest and abdominal CT scans are performed every 6–12 months during the first three years, and then annually up to five years. Pan-colonoscopy is scheduled at one year, followed by three years, and subsequently every five years.

## 4. Endoscopic Treatment

**Figure 1:**
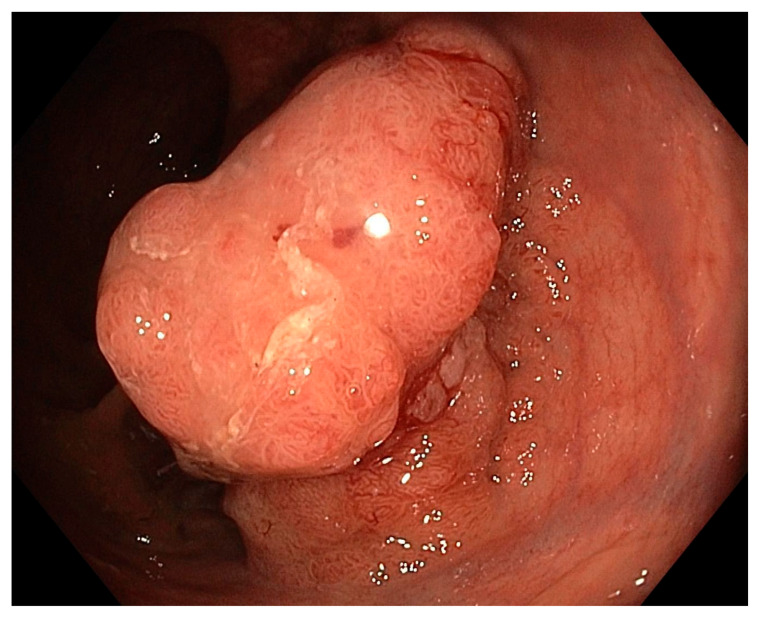
Small rectal lesion suitable for endoscopic curative resection.

Endoscopic therapy for rectal cancer refers to treatment using a flexible endoscope (typically up to 15 mm in diameter of distal end) inserted transanally. The benefit of flexible endoscopy lies in its relative ease of performance without the routine need for general anaesthesia. Endoscopic techniques preserve the rectum as the target organ, which favourably impacts both the early post procedure course and long-term functional outcomes. The limitation of endoscopic techniques, naturally, is the inability to remove and examine regional lymph nodes. Consequently, endoscopic therapy targets T1 colorectal cancer (with shallow invasion into the submucosa) for which the risk of lymphatic metastases is low, or relatively low compared to surgical morbidity and mortality (Figure 1). Balancing these risks is, of course, individualized based on the patient’s overall condition and comorbidities.

Endoscopy holds a nearly dominant position in the endoscopic treatment of neoplastic lesions at the adenoma stage, including those with high-grade dysplasia [38]. This group also includes Tis adenocarcinoma (intramucosal carcinoma or carcinoma invading into the lamina muscularis mucosae), since the risk of lymphatic metastases is zero in these colorectal lesions.

Endoscopic diagnosis of T1 rectal cancer is based on macroscopic assessment of lesion location, size and morphology (Paris classification and derived LST—laterally spreading tumor classification), combined with detailed visualization using chromoendoscopy (today mostly virtual) and magnification (most frequently used JNET classification—Japanese NBI expert). This precise diagnostic approach enables us, on one hand, to define lesions unsuitable for endoscopic treatment (JNET 3), and on the other hand, to identify lesions with a low probability of invasive carcinoma (e.g., homogeneous granular type LST, JNET 2a) [38].

Prior to an endoscopic procedure for a suspected invasive rectal carcinoma, we recommend staging in a manner similar to surgical staging: CT mainly to rule out distant metastases and pelvic MRI to assess lymphadenopathy. MRI for T-staging of lesions is not routinely used because accuracy for T2 versus T1 is relatively low (despite new feature of submucosal enhancing strip) [39]. Rectal endoscopic ultrasound shows only slightly better potential at this point [40].

### 4.1. Endoscopic Mucosal Resection (EMR)

EMR is considered the conventional technique for removing colorectal neoplastic lesions—the technique uses metal snares, either with electrocautery (hot-snare) or without (cold-snare) [38], typically with submucosal injection to lift the lesion, or occasionally without injection using water immersion (underwater EMR) [41]. The problem with EMR for T1 rectal carcinoma lies in its lower control of lateral resection margin. Capacity for en bloc and R0 resection is lower in comparison with other techniques, quality of the specimen is thus reduced and reliable histological assessment compromised.

Therefore, upon endoscopic suspicion of invasive rectal carcinoma, one of the following techniques is indicated to achieve en bloc resection with better lateral and vertical margin control. Recent ESGE guidelines recommend for rectal lesions over 20 mm in diameter with dominant nodules bigger than 10 mm and features of submucosal invasion to consider endoscopic submucosal dissection instead of EMR [38].

### 4.2. Endoscopic Submucosal Dissection (ESD)

**Figure 2:**
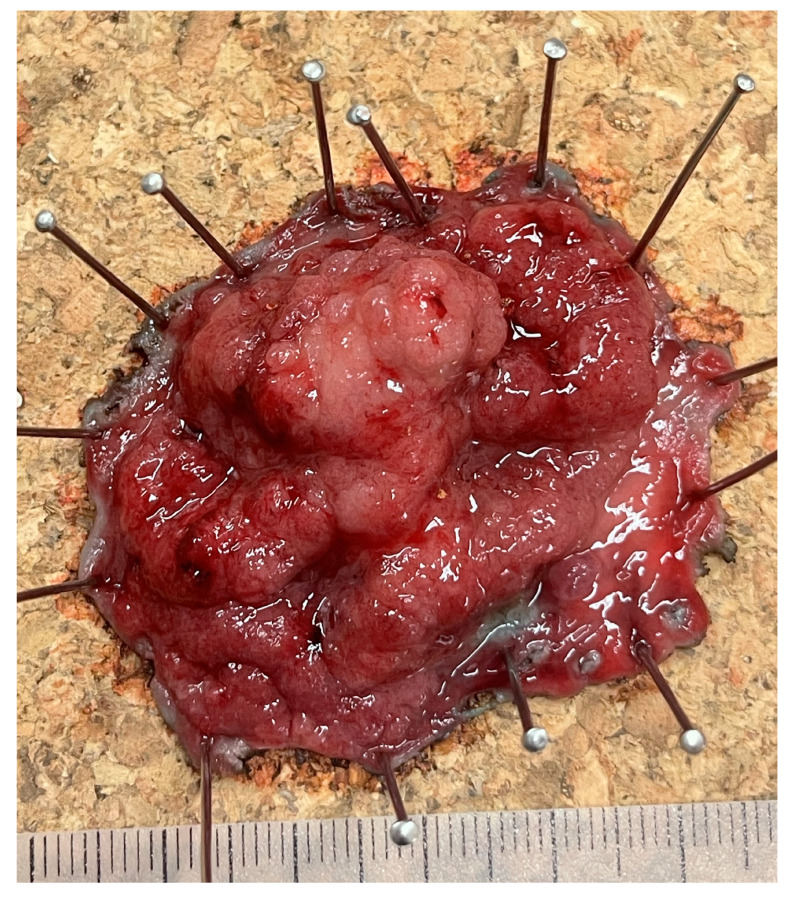
Rectal cancer removed by endoscopic submucosal dissection.

This technique also uses submucosal injection to lift the lesion, but unlike EMR, it employs targeted dissection in the submucosa using various types of endoscopic micro knives to separate the mucosa containing the tumor. The aim is en bloc resection of the lesion, regardless of size (even circular dissection is possible) (Figure 2). Recently, various traction methods (tunnel method, pocket method, traction with thread, rubber band, clips, etc.) have been used to facilitate the procedure [42].

Several factors lead to the tendency to dissect deeper than the submucosa: (1) Some tumors, although superficially invading the submucosa, induce fibrosis that poses a technical obstacle to ESD; (2) isolated deep submucosal invasion is a relatively weak risk factor for distant metastases; (3) the higher risk of distant disease with deep submucosal invasion is offset in some patients by a high operative risk. Thus, newer techniques enabling resection including the lamina muscularis propria are being employed: endoscopic full-thickness resection (eFTR) and endoscopic intermuscular dissection (EID).

### 4.3. Endoscopic Full-Thickness Resection (eFTR)

This uses instrumentation based on the OTSC (over-the-scope clip) system, which first “ligates” the tissue drawn into the endoscope cap using graspers, and then resects it with an electrocautery snare [43,44]. This is suitable for lesions up to 15 mm (maximum 20 mm) in diameter. Resected specimen covers the full thickness of rectal wall.

### 4.4. Endoscopic Intermuscular Dissection (EID)

This technique takes advantage of the anatomy of the rectum—unlike the colon, it has an internal circular and external longitudinal muscularis propria layer separated by loose connective tissue. The technique is similar to ESD, but the dissection plane (at least in part of the procedure) is not the submucosal but the intermuscular connective tissue layer [45]. This allows acquisition of full submucosal specimens while preserving the outer muscularis layer (avoiding open perforation into perirectal tissue) [46]. Traction methods are also used in this type of dissection like tunneling [47] or tractions [48].

A comparison of the different endoscopic techniques can be made based on (1) achievement of en bloc and R0 resection, (2) risk of local recurrence, (3) procedure duration and difficulty, and (4) risk of complications (primarily perforation). From these criteria, the fastest method is EMR, which is, however, limited to lesions of about 20 mm because beyond this size en bloc resection is no longer achievable (compromising histological assessment and increasing local recurrence risk). Similarly, eFTR is limited by lesion size but has the advantage of full-thickness resection and relative ease of procedure. ESD and EID are the most technically demanding, with higher perforation risk but high rates of en bloc and R0 resections and low local recurrence rates [49].

### 4.5. Assessment of Curative Resection

Assessment of whether an endoscopic resection is curative is based on estimating the risk of distant metastasis according to histologic risk factors. The risk of distant metastasis is then weighed against the morbidity and mortality of potential salvage surgery. Individual risk factors do not carry equivalent weight. Major negative prognosticators include lympho-vascular invasion (OR 6), tumor budding (OR 5), high tumor grading (G3) (OR 3,6) and submucosal invasion ≥ 1000 µm (OR 2,8) [50]. The accumulation of risk factors increases the risk of distant metastasis more than the simple sum of risks. Conversely, if submucosal invasion depth exceeds the threshold as an isolated factor, the risk of lymphatic metastases remains less than 3% [50].

If the risk of distant metastasis significantly exceeds the individual surgical risk, salvage rectal resection is indicated. Another possible strategy in case of a high risk of distant metastasis after endoscopic resection is adjuvant therapy [51].

### 4.6. Post-Endoscopic Surveillance

Surveillance after endoscopic therapy is similar to that following other rectal cancer treatment modalities: the first postoperative colonoscopy within one year of treatment, the next at three years, and then at five years, continuing while benefit persists. With increased risk of local recurrence, additional rectoscopy in the first 2–3 years at 3–6-month intervals is included [52].

## 5. Surgical Treatment

Surgery remains the cornerstone of curative treatment for rectal cancer. The gold standard continues to be rectal resection with total mesorectal excision (TME), as first described by Heald in 1986 [53]. This technique is based on precise dissection of the mesorectum along its embryological fascial plane, ensuring complete removal of lymphatic structures and minimizing the risk of local recurrence. Since its original description, the technique has undergone significant refinement, particularly due to the introduction of minimally invasive approaches, advancements in imaging modalities, and development of surgical tools such as staplers and robotic systems.

Nowadays, rectal resections are primarily performed laparoscopically or robotically, minimizing surgical invasiveness while maintaining oncological radicality. The oncological equivalence of laparoscopic surgery to open surgery has been confirmed in randomized trials, such as the COLOR II study [54], which demonstrated comparable oncologic outcomes with reduced perioperative morbidity. Another technical innovation expanding surgical options is transanal total mesorectal excision (TaTME), which provides optimal access to the distal rectum—particularly advantageous in patients with a narrow pelvis or bulky tumors.

In addition to patients with localized tumors, selected patients with stage IV disease may also undergo surgery with curative intent. These typically involve cases with resectable liver or lung metastases. Three main therapeutic sequencing strategies are available: the “bowel-first” approach (primary resection of the rectal tumor), the “liver-first” approach (initial resection of liver metastases), and simultaneous resection of both the rectal tumor and metastases. The choice of optimal strategy depends on tumor biology, extent of metastatic disease, response to systemic therapy, and the patient’s overall condition [55].

Surgical strategy is also guided by the clinical stage of the disease according to the TNM classification. Patients with stage I disease are typically managed with primary resection without prior oncologic treatment. In contrast, those with stage II or III tumors usually receive preoperative (neoadjuvant) chemoradiotherapy aimed at tumor downsizing, reducing microscopic spread (downstaging), and improving resectability [56]. Surgical resection follows 6–12 weeks after completion of neoadjuvant therapy. In patients who have undergone endoscopic tumor resection but fail to meet histopathological criteria for radical excision, completion of surgical resection is indicated. These criteria include an R1 resection margin (<1 mm from the resection edge), lymphatic, vascular, or perineural invasion, and high tumor grade (G3 or G4) [57].

### 5.1. Low Anterior Resection

The standard surgical procedure remains anterior resection, or low anterior resection (LAR) for low-lying tumors, with total mesorectal excision. The procedure begins with lateral mobilization of the sigmoid colon, proceeding distally to the splenic flexure. During this phase, the inferior mesenteric artery (IMA) and inferior mesenteric vein (IMV) are exposed, and some authors prefer a high ligation of the vascular pedicle. However, studies have not demonstrated a clear oncological advantage of high ligation compared to ligation at the origin of the superior rectal artery (SRA) [58]. Mobilization of the descending colon and sigmoid is performed medially to laterally along the avascular Toldt’s fascia. TME begins at the promontory and continues laterally along the embryological planes down to the pelvic floor. During dissection, it is crucial to protect the left ureter and hypogastric nerves to minimize the risk of urinary and sexual dysfunction. Ventral dissection proceeds along Denonvilliers’ fascia, which separates the rectum from the prostate and seminal vesicles in men, or from the posterior vaginal wall in women.

**Figure 3:**
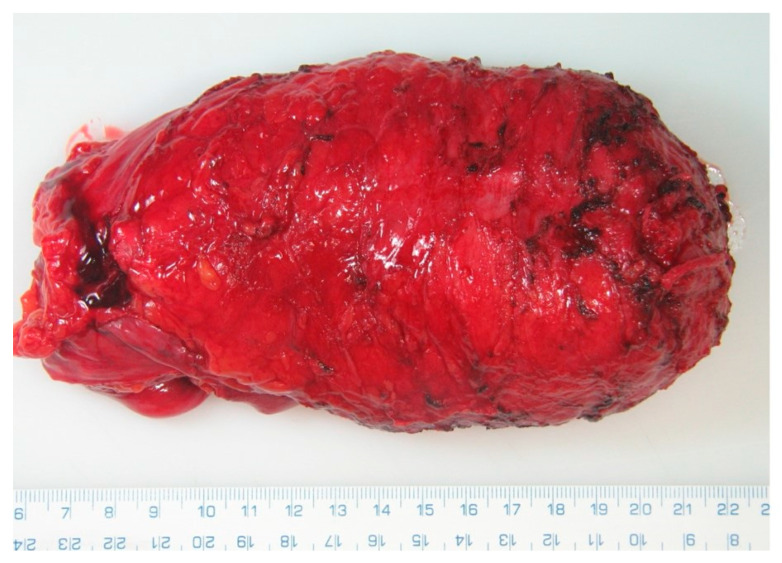
Representative specimen of rectum with TME.

**Figure 4:**
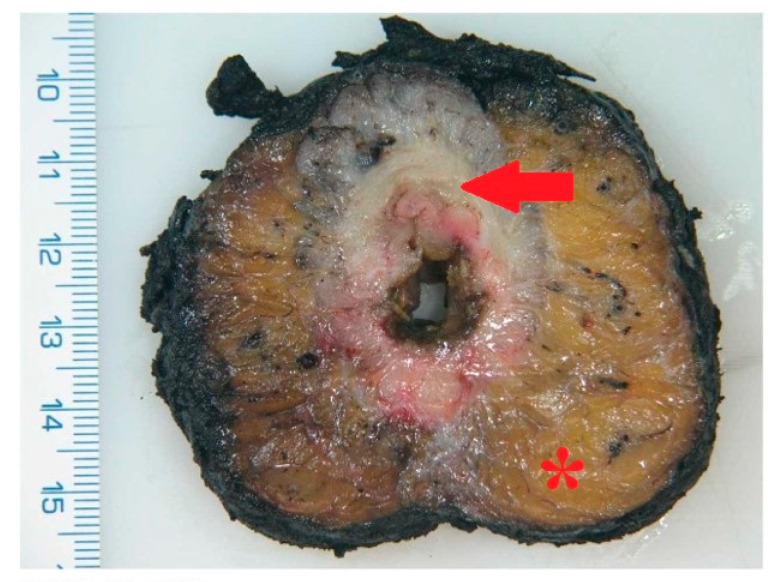
Pathological specimen after cutting—* = mesorectum, red arrow = tumor.

Upon completion of the resection, the specimen is sent for histopathological analysis (Figure 3 and Figure 4). Bowel continuity is most often restored via a stapled end-to-end anastomosis. In cases with high risk of anastomotic leakage, a protective ileostomy is preferred, or alternatively, a Hartmann’s procedure with end colostomy may be performed.

In situations where sphincter preservation is not feasible—such as with very low-lying tumors—abdominoperineal resection (APR, Miles’ operation) or intersphincteric resection (ISR) is indicated. ISR is performed in two phases. The abdominal phase involves mobilization of the sigmoid colon and transection of the bowel, followed by rectal dissection to the pelvic floor. The patient is then repositioned prone for the perineal phase, during which the entire rectum including the anal canal and sphincter complex is excised. In ISR, the external sphincter is preserved while the internal sphincter is selectively excised. The rectum is detached from the pelvic floor, which is then reconstructed using sutures or a prosthetic implant, depending on local anatomy. Patients undergoing ISR typically have lower resting sphincter pressure and thus higher Wexner scores compared to LAR; however, studies show that continence tends to improve over time [59].

### 5.2. Pelvic Exenteration

Pelvic exenteration is an extremely radical surgical procedure reserved for patients with locally advanced or recurrent rectal cancer where other therapeutic options fail to provide oncological control. The primary objective is to achieve complete tumor excision (R0), which is a key determinant of long-term survival [60,61].

Posterior pelvic exenteration is indicated for tumors infiltrating the uterus or posterior vaginal wall, and involves abdominoperineal rectal amputation, hysterectomy with adnexectomy, and resection of the posterior vaginal wall.

Total pelvic exenteration is performed in cases involving both the anterior and posterior compartments and entails removal of all pelvic organs. The resection phase includes rectal amputation, cystectomy, and excision of reproductive organs—prostatectomy and vesiculectomy in men, hysterectomy with adnexectomy and colpectomy in women. In composite total exenteration, the coccyx and, if necessary, part of the sacrum are also resected.

Supralevator exenteration is indicated for involvement of the upper rectum, allowing preservation of the pelvic floor and sufficient rectal stump for colorectal or coloanal anastomosis. In contrast, infralevator exenteration includes resection of the pelvic floor musculature.

Recent systematic reviews report R0 resection rates of approximately 70–82% in patients with primary locally advanced rectal cancer and slightly lower rates in recurrent cases [61,62]. Five-year overall survival ranges from 29% to 78%, with significantly better outcomes in patients with primary tumors and negative margins [60]. Despite good oncologic outcomes, the procedure has considerable impact on patients’ quality of life and psychological well-being. In the reconstructive phase, urinary and fecal diversion is required—most commonly via stoma and conduit formation. High rates of sexual dysfunction are observed, and in women of reproductive age, premature menopause may occur. Nevertheless, studies suggest a rapid improvement in quality of life as early as two months postoperatively, with continued improvement over the first year after surgery [63,64].

### 5.3. Transanal Total Mesorectal Excision (TaTME)

A relatively new approach is transanal total mesorectal excision (TaTME), systematically described by Lacy in 2010 [65]. This technique is primarily indicated for low-lying tumors where traditional abdominal access may not provide adequate visualization and resection precision. Advantages of TaTME include better access to the lower rectum, higher quality of mesorectal excision, and a lower risk of autonomic nerve injury [66]. The procedure can be performed purely transanally or in combination with laparoscopic or robotic access.

A recent meta-analysis comparing robotic TME and TaTME found that the only significant advantage of the robotic approach was a higher rate of complete mesorectal excision, while other postoperative outcomes and complication rates were comparable between the two techniques [67]. Another meta-analysis comparing the laparoscopic approach with TaTME showed a lower rate of anastomotic leakage in the laparoscopic group; however, TaTME was associated with a reduced incidence of permanent colostomy. Other perioperative outcomes were similar between the two approaches [68].

Despite comparable postoperative outcomes and potential benefits, international registries have also highlighted the risks associated with this technique and therefore recommend that TaTME be restricted to high-volume centers with structured training programs and careful patient selection [69].

### 5.4. Transanal Endoscopic Microsurgery (TEM)

In 1983, Buess et al. reported the use of transanal endoscopic microsurgery (TEM) in 140 patients with rectal lesions, 30 of whom had rectal cancer [70]. This technique employs a specially designed rigid rectoscope through which instruments and optics are introduced. At the time, this was a revolutionary method and is still used selectively at some centers today. Since complete tumor resection is required, TEM is best suited for removal of smaller lesions with low-risk features, for which it offers favorable postoperative outcomes [71].

### 5.5. Transanal Minimally Invasive Surgery (TAMIS)

Introduced in 2010, transanal minimally invasive surgery (TAMIS), represents an evolution of local excision techniques and is suitable for selected patients with early-stage rectal cancer. Compared to TEM, TAMIS has advantages in terms of accessibility, lower equipment costs, and easier adoption due to the use of standard laparoscopic instruments and laparoscopic single-incision port inserted into the anus [72]. Indications for TAMIS include well to moderately differentiated T1–T2 tumors without lymphovascular or perineural invasion, mucinous components, or lymphadenopathy on staging investigations. The lesion should not exceed 3 cm in size, involve less than one-third of the rectal circumference, be non-fixed to the rectal wall, and have an anticipated clear resection margin. Comparative studies between TEM and TAMIS show similar outcomes in terms of complete resection and oncological efficacy [73]. When compared to endoscopic techniques, both TEM and TAMIS offer equivalent results and represent promising organ-preserving options with good oncologic outcomes in selected patients [74]. Other advantages of minimally invasive approaches are better access to lesions in lower rectum, where endoscopic resection can be technically challenging, and possibility to perform the procedure under spinal anesthesia.

## 6. Summary

The management of rectal cancer has undergone substantial evolution, transitioning from a one-size-fits-all surgical paradigm to a nuanced, multidisciplinary approach emphasizing oncologic safety, organ preservation, and quality of life. While total mesorectal excision (TME) remains the gold standard for curative resection, its role is increasingly complemented by advanced endoscopic techniques and minimally invasive surgical strategies. Innovations such as endoscopic submucosal dissection (ESD), endoscopic intermuscular dissection (EID), and transanal total mesorectal excision (TaTME) offer promising avenues for selected patients, particularly those with early-stage disease or significant comorbidities.

A growing body of evidence suggests that neoadjuvant therapy—particularly chemoradiotherapy—may represent overtreatment in a subset of patients, especially those with favorable T3 tumors lacking high-risk features. This highlights an urgent need for improved risk stratification tools, including imaging biomarkers and molecular profiling, to avoid unnecessary toxicity and optimize patient outcomes. Additionally, current staging modalities remain limited in their ability to predict lymph node involvement or accurately assess complete response following neoadjuvant treatment, creating uncertainty in treatment planning.

This narrative review emphasizes the critical importance of personalized, evidence-based treatment strategies in rectal cancer care. By contextualizing surgical and non-surgical options within a modern multidisciplinary framework, it contributes to the ongoing scholarly discussion about how best to balance oncologic radicality with functional preservation in the era of precision oncology.

## 7. Conclusions

Rectal cancer care is moving from stage-based, procedure-driven pathways to biologically informed, goal-concordant strategies. The central task is no longer choosing “surgery vs. non-surgery,” but matching the intensity and sequence of therapies to an individual tumor’s risk while preserving function. This review integrates endoscopic, surgical, and systemic options into a risk-adaptive framework that prioritizes organ preservation when oncologically safe and escalates treatment when adverse biology is present (e.g., EMVI, threatened CRM, lateral nodes, poor differentiation). We argue that routine neoadjuvant chemoradiotherapy for all cT3 tumors is unlikely to remain tenable; instead, selection should be guided by composite clinical–radiologic–molecular profiles and by patient-reported priorities.

Key challenges now are to (1) validate de-escalation for favorable T3 disease and formalize criteria for watch-and-wait after clinical complete response; (2) standardize response assessment (MRI and endoscopic criteria, biopsy policies including liquid biopsy) and long-term surveillance; (3) define where advanced endoscopy (ESD/EID/eFTR) can substitute for radical surgery without compromising nodal control; (4) integrate ctDNA and MSI/MMR status to tailor neoadjuvant and adjuvant therapy; (5) clarify indications, training, and quality metrics for TaTME and other complex techniques; and (6) embed quality-of-life and functional outcomes as co-primary endpoints in trials. Progress on these fronts—supported by pragmatic multicenter studies, real-world registries, and AI-driven prediction—will enable truly personalized, value-based rectal cancer management.

## Figures and Tables

**Table 1:** Comparison of TNT vs. Standard CRT.

Parameter	TNT	Standard CRT
**Timing of Chemotherapy**	Sequential: before or after radiotherapy	Concurrent with radiotherapy
**Treatment Components**	Radiotherapy (SCRT/LCRT) + full systemic chemotherapy (FOLFOX/CAPOX)	Radiotherapy (mostly LCRT) + fluoropyrimidine (capecitabine/5-FU)
**Pathological Complete Response (pCR)**	Higher: 23–28%	Lower: 10–15%
**Micrometastatic Disease Control**	Significantly better	Limited
**“Watch-and-Wait” Strategy**	Significantly higher (OPRA: up to 50–60%	Rare
**Preoperative Duration**	Longer: 3–6 months	Shorter: 6–8 weeks
**Toxicity**	Higher (hematologic, GI)	Lower systemic, higher local (proctitis, dermatitis)
**Guidelines Recommendation**	Preferred for high-risk LARC (ESMO, ASTRO, NCCN)	Acceptable for low/intermediate-risk or TNT-intolerant

**Table 2:** Summary of key TNT trials.

Study	TNT Regimen	3–5 Year DFS (%)	Comparison with Standard CRT
**RAPIDO [13]**	SCRT + 6–9 cycles CAPOX/FOLFOX	76.3 vs. 69.6	*p* = 0.01
**PRODIGE-23 [12]**	FOLFIRINOX + chemoradiation	76 vs. 69	*p* = 0.03
**STELLAR [15]**	SCRT + CAPOX	64.5 vs. 62.3	No OS difference, higher toxicity

## Abbreviations

The following abbreviations are used in this manuscript:

CRC	Colorectal Cancer
HDI	Human Development Index
EOCRC	Early Onset Colorectal Cancer
CT	Computed Tomography
MRI	Magnetic Resonance Imaging
TNT	Total Neoadjuvant Therapy
cCR	Clinical Complete Response
pCR	Pathological Complete Response
SCRT	Short-course Radiotherapy
LCRT	Long-course Radiotherapy
CRT	Chemoradiotherapy
TME	Total Mesorectal Excision
dMMR	Deficient Mismatch Repair
MSI	Microsatellite instability
LST	Laterally Spreading Tumor
EMR	Endoscopic Mucosal Resection
ESD	Endoscopic Submucosal Dissection
eFTR	Endoscopic Full Thickness Resection
EID	Endoscopic Intermuscular Dissection
TaTME	Transanal Mesorectal Excision
LAR	Low Anterior Resecion
ISR	Intersphincteric Resection
TEM	Transanal Endoscopic Microsurgery
TAMIS	Transanal Minimally Invasive Surgery
